# Cholinergic Urticaria: A Case Report

**DOI:** 10.7759/cureus.30869

**Published:** 2022-10-30

**Authors:** Luis Carlos Fonseca, Cláudia Rodrigues, Antonio Jose Lemos

**Affiliations:** 1 General and Family Medicine, USF Grão Vasco, Viseu, PRT

**Keywords:** primary care, inducible urticaria, case reports, urticaria, skin diseases, allergy and immunology

## Abstract

Physical activity plays an important role in our health. Yet, the practice of physical exercise or the consequent sweating itself can be a causal factor of skin pathology. Cholinergic urticaria is an entity characterized by itchy papules associated with heat and/or sweat, with exercise being the main triggering factor. While urticaria can be common, cholinergic urticaria is a rare entity in primary care and is usually overlooked. Promoting medical literacy and public awareness should be incorporated as a tool to provide support and management for these cases, which is emphasized in this case presentation.

This case report presents a typical case, and discusses the added value of new technologies, like smartphones, in the diagnosis of cholinergic urticaria, its symptomatic treatment with antihistamines, and its follow-up. Patient empowerment and reassurance are the key aspects of therapeutic success and the role of the family doctor is crucial. The physician in this report reassured the patient, by properly explaining her condition, possible limitations, and when to seek medical attention, which resulted in a considerable improvement in her quality of life.

## Introduction

Physical exercise is an important tool in the therapeutic arsenal of physicians. Its benefits, either in the prevention or the management of specific pathologies, represent a unique added value. However, there are pathologies triggered by physical exercise itself, which can have potentially life-threatening symptoms, such as exercise-induced anaphylaxis [1-4]. In a more benign but closely related context, this article addresses a case of cholinergic urticaria in a young woman observed in primary care.

Cholinergic urticaria, a rare and usually overlooked entity in primary care, is a type of chronic inducible urticaria and may account for only 1% of reported cases of urticaria [4-9]. It involves relapsing episodes of skin lesions, as in all urticaria types, with varying sizes as well as associated swelling and erythema. Symptoms of pruritus or burning are also common [4-11]. The vast majority of cases resolve within a few hours and may or may not be associated with angioedema [4-6].

The pathogenesis of this condition is still not completely understood; it might be triggered by an increase in body temperature, hypersensitivity to certain elements of sweat, or other causes [4-6,11]. Antihistamines are the main treatment option but other therapeutics may also be needed [12-13]. Patient expectations can prove to be a challenge, which reinforces the importance of an adequate follow-up by the family doctor.

## Case presentation

The patient was a 31-year-old female with a medical history of depressive syndrome, currently on a daily intake of fluoxetine 20 mg, and topiramate 100 mg twice daily. There was no record of any known food, drug, or other allergies. On the patient's initial appointment at a primary healthcare center, the complaint was recurrent exanthema associated with mild pruritus with about two months of evolution, which always had a sudden onset. The patient was mainly concerned that the symptoms could worsen or become life-threatening.

She associated the appearance of the rash with moderate physical activity and reported that it was also triggered by any activity that led to sweating, namely stressful situations or prolonged sun exposure. For example, high-intensity running for more than 100 meters. Lesions appeared in less than five minutes, and the more intense the stimulus, the earlier the onset was. It predominated in the upper limbs and later spread throughout the body in a matter of minutes. It resolved gradually and spontaneously in about one to two hours, with the interruption of physical exercise or activity observed to be triggering the symptoms.

She denied any respiratory complaints, such as dyspnea, cough, wheezing, and laryngeal complaints. She also denied any similar symptoms when in the bath, regardless of the water temperature. The patient also denied any family or personal history of atopy, hyperhidrosis, and respiratory, dermatological, or autoimmune diseases. She had self-initiated multivitamin supplementation rich in carotenes, without any improvement of complaints presented.

**Figure 1:**
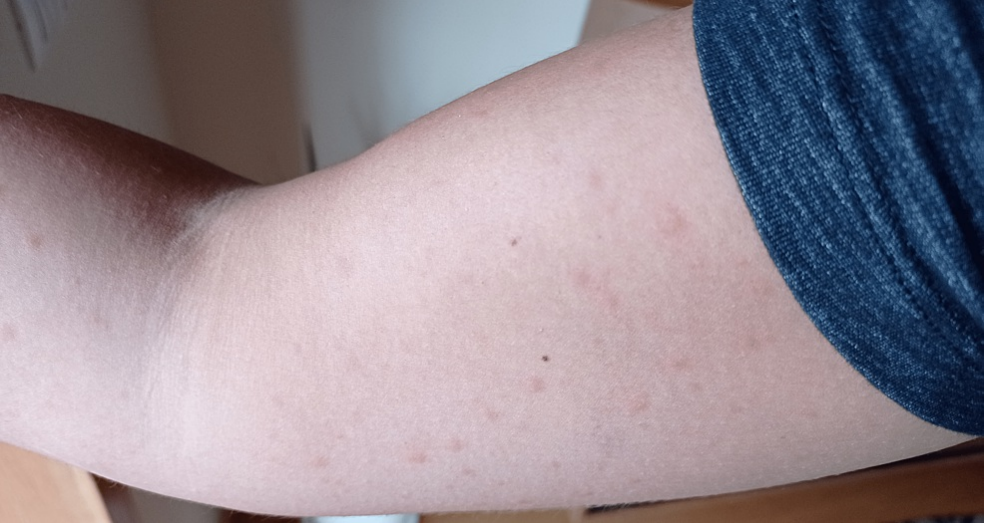
Arm urticaria lesions Photo provided by the patient with the consent to publish

**Figure 2:**
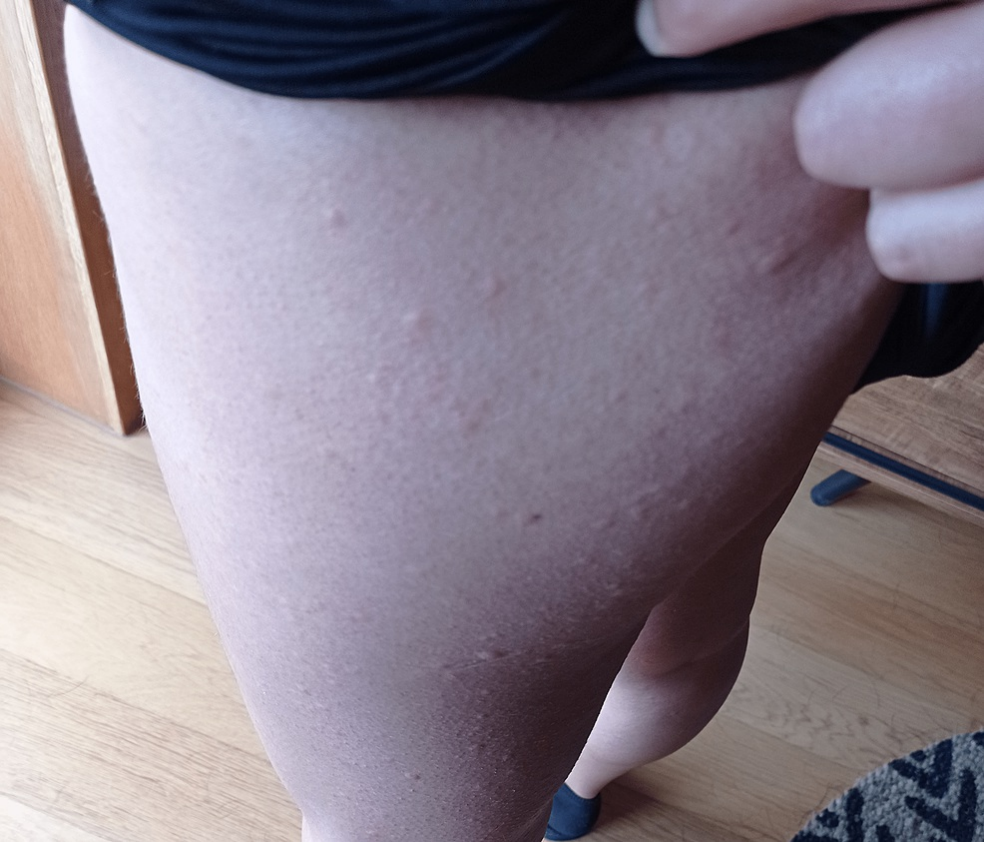
Thigh urticaria lesions Photo provided by the patient with the consent to publish

No lesions were observed at the medical appointment, but the patient showed photographs of them (Figures 1, 2) - papules of various sizes, reddened, predominantly on the trunk and limbs but dispersed throughout the body, including the palms of the hands, soles of the feet, and scalp.

The rest of the examination, namely pulmonary auscultation and search for adenopathies, did not reveal any other alterations. There was no recent weight loss and the qualitative serological test employed for screening of allergic sensitization had returned negative a year ago.

With the clinical features suggestive of cholinergic urticaria, she was medicated with bilastine 20 mg daily, with partial improvement in the symptom intensity and prolongation of latency time in the appearance of lesions.

On her own initiative, she was observed by an immunoallergy specialist, who agreed with the diagnosis of cholinergic urticaria, and recommended, in addition to the daily intake, the prophylactic use of bilastine, 30-60 minutes before the initiation of the potential triggering factor, with the possibility of increasing intake frequency up to three times a day. No other complementary diagnostic tests were performed, which reinforced the importance of a correct explanation of the patient’s condition. He told her about the possibility that the urticaria might stop spontaneously within months to years.

Currently, after 16 months, the patient still takes both daily and prophylactic bilastine 20 mg before exercise. The lesions take about 30-40 minutes to appear, depending on the intensity of the continuous physical activity, and there is no associated pruritus.

## Discussion

The discussed case, although uncommon, represents a typical manifestation of the referred pathology based on what is described in the literature [4-11]. In these cases, carrying out complementary diagnostics like a quantitative measurement that corroborates the clinical diagnosis can be challenging and it presents a serious limitation. In our patient, since exercise was the main trigger, we could always perform a provocation test to confirm the appearance of skin lesions as well as exclude other entities, but the anamnesis was sufficient [4].

Faced with a condition with more than six weeks of evolution, we were dealing with a case of chronic urticaria [4]. As there was an apparent triggering factor, it was definitely a case of inducible urticaria. Regarding the differential diagnosis, the patient mentioned prolonged sun exposure as a trigger, however, only with associated sweating. She also denied asymmetric distribution of early lesions while driving a car that could be related to asymmetric direct sun exposure, which could indicate solar urticaria. She also denied the influence of air or water temperature in triggering the symptoms, making cold or heat urticaria an unlikely diagnosis [4].

Cholinergic urticaria is a type of urticaria characterized by papules associated with heat, and its pathogenesis is still not completely understood [4-6,9]. The current hypothesis states that it is triggered by an increase in body temperature due to exercise, consumption of spicy foods, or other stressful factors. It may also be related to a hypersensitivity to certain elements of sweat. Some studies have attributed this sensitivity to the acetylcholine released [6,11].

Although not life-threatening, it can be a source of serious discomfort with a consequent decrease in the quality of life. It can even affect the practice of physical exercise and lead to the avoidance of socialization due to the fear of potentially stressful situations, which highlights the importance of patient empowerment. We counseled our patient about the benignity of her condition, and told her that she must go out and live her normal life; however, we also explained to her the warning signs that should make her seek medical attention.

These lesions consist of multiple small papules, between 1 and 5 mm in size, which can converge and persist for minutes to hours [4-6,10]. Their distribution on the body is homogeneous, with the trunk and upper extremities predominantly affected [5-6]. The main complaint is pruritus, which may be accompanied by pain or skin sensitivity. Only a small percentage of patients may experience symptoms such as dizziness and chest tightness [4-5,9]. Our patient presented with the typical and common manifestations mentioned above.

There is a lot of discrepancy in studies concerning the type of population affected by this condition [5-8]. According to some studies, there is a predominance of males, and the average age of incidence is between the second and third decades of life. A considerable percentage has atopy as comorbidity and about 50% may have seasonal aggravation, not specific to a given season (some in summer, others in winter).

In the vast majority of cases, it is a self-limiting disease, which ceases, on average, after about 48 months. Treatment consists of antihistamines for symptomatic and prophylactic management (prior or not to physical exercise) [4-11]. Omalizumab represents a potential second-line therapy, which is already being used; however, there is still no specific evidence about its efficacy [12-13]. Our patient's symptoms were controlled with the first-line treatment despite some lesions with more intense exercise, but she does not need to stop the activity.

## Conclusions

This report discussed an uncommon entity that is encountered in primary care. Nevertheless, physicians must be aware of this clinical entity in order to reassure the patients and ensure a correct approach toward managing it, since clinical manifestations and patient expectations can have a great impact on their quality of life. Promoting medical literacy and public awareness represents an important tool in dealing with these cases. Nowadays, technology can be a valuable asset in the assessment of patients' complaints with the possibility of taking photos and creating written systematized records; however, the overload of information available online can create many doubts or cause delays in seeking medical assistance. In this case, the diagnosis and subsequent management were only possible with the aid of the photos taken by the patient.

The patient's physician properly reassured her and she is now quite capable of managing her situation. She knows that her condition is not life-threatening, that she can exercise, and is aware as to which symptoms should merit concern. This was achieved by appropriately counseling the patient as well as clarifying her doubts. The follow-up by our medical team enabled her to improve her quality of life. However, she still has expectations for a total resolution in the short term, as this situation affects her on a daily basis, which reveals the importance of maintaining an adequate follow-up.

## References

[1] Christensen MJ, Eller E, Kjaer HF, Broesby-Olsen S, Mortz CG, Bindslev-Jensen C (2019). Exercise-induced anaphylaxis: causes, consequences, and management recommendations. Expert Rev Clin Immunol.

[2] Feldweg AM (2015). Exercise-induced anaphylaxis. Immunol Allergy Clin North Am.

[3] Giannetti MP (2018). Exercise-induced anaphylaxis: literature review and recent updates. Curr Allergy Asthma Rep.

[4] Godse K, De A, Zawar V, Shah B, Girdhar M, Rajagopalan M, Krupashankar DS (2018). Consensus statement for the diagnosis and treatment of urticaria: a 2017 update. Indian J Dermatol.

[5] Kim JE, Eun YS, Park YM (2014). Clinical characteristics of cholinergic urticaria in Korea. Ann Dermatol.

[6] Geller M (2020). Clinical management of exercise-induced anaphylaxis and cholinergic urticaria. J Allergy Clin Immunol Pract.

[7] Pozderac I, Lugović-Mihić L, Artuković M, Stipić-Marković A, Kuna M, Ferček I (2020). Chronic inducible urticaria: classification and prominent features of physical and non-physical types. Acta Dermatovenerol Alp Pannonica Adriat.

[8] Montgomery SL (2015). Cholinergic urticaria and exercise-induced anaphylaxis. Curr Sports Med Rep.

[9] Fukunaga A, Washio K, Hatakeyama M, Oda Y, Ogura K, Horikawa T, Nishigori C (2018). Cholinergic urticaria: epidemiology, physiopathology, new categorization, and management. Clin Auton Res.

[10] Abajian M, Schoepke N, Altrichter S, Zuberbier T, Maurer M (2014). Physical urticarias and cholinergic urticaria. Immunol Allergy Clin North Am.

[11] Tokura Y (2016). New etiology of cholinergic urticaria. Curr Probl Dermatol.

[12] Ghazanfar MN, Holm JG, Thomsen SF (2020). Omalizumab for cholinergic urticaria: 6 months prospective study and systematic review of the literature. Dermatol Ther.

[13] Chicharro P, Rodríguez P, de Argila D (2017). Omalizumab in the treatment of chronic inducible urticaria. Actas Dermosifiliogr.

